# Resveratrol inhibits ferroptosis and decelerates heart failure progression via Sirt1/p53 pathway activation

**DOI:** 10.1111/jcmm.17874

**Published:** 2023-07-24

**Authors:** Wei Zhang, Shaohuan Qian, Bi Tang, Pinfang Kang, Heng Zhang, Chao Shi

**Affiliations:** ^1^ Department of Cardiovascular Medicine The First Affiliated Hospital of Bengbu Medical College Bengbu City China; ^2^ Department of Cardiac Surgery The First Affiliated Hospital of Bengbu Medical College Bengbu City China

**Keywords:** ferroptosis, heart failure, resveratrol

## Abstract

Resveratrol is an organic compound widely studied for its therapeutic uses. We investigated whether resveratrol exerts cardioprotective effects by inhibiting ferroptosis via the Sirt1/p53 pathway. A heart failure model was established by aortic coarctation in Sirt1 knockout mice. The superoxide dismutase (SOD), glutathione (GSH) levels and mitochondrial morphology in murine heart tissues were assessed at different time points to determine the role of ferroptosis in heart failure progression. The cardiac function of mice with heart failure was evaluated by determining the brain natriuretic peptide (BNP) and sST2 concentration and conducting echocardiography. Human induced pluripotent stem cell‐derived cardiomyocytes (hiPSC‐CMs) were transfected with the p53 K382R mutant and Sirt1 interference lentiviral vectors. Immunoprecipitation (IP) experiments were performed to investigate whether Sirt1 influences ferroptosis via p53 K382 acetylation and SLC7A11 expression modulation. Resveratrol improved cardiac function in mice and decelerated ferroptosis and fibrosis progression in heart failure. However, the ability of resveratrol to prevent ferroptosis and treat heart failure was lost after silencing Sirt1. Sirt1 reduced ferroptosis by diminishing the levels of p53 K382 acetylation, reducing the degradation of SLC7A11, and increasing the levels of GSH and glutathione peroxidase 4 (GPX4) in cells. In conclusion, by activating the Sirt1/p53 pathway in heart failure, resveratrol decreased the depletion of SLC7A11, inhibited ferroptosis, and improved cardiac function.

## 1 INTRODUCTION

Cardiac remodelling, driven by inflammatory responses, neurohormones and reactive oxygen species (ROS) accumulation, exacerbates cardiac fibrosis and enlargement.^1^, ^2^ Due to the abnormal function and structure of the heart, the ventricular ejection function is reduced, which in turn causes organ congestion and ischemia.^3^, ^4^ Currently, epidemiological studies show that the incidence of heart failure in adults is approximately 2%. As age continues to increase, heart failure incidence increases from 1% at the age of 55 to 10% at the age of 70.^5^, ^6^ Thus, systemic inflammation and oxidative stress inhibition are crucial for the prevention of myocardial fibrosis and heart failure progression.

Ferroptosis is closely related to cardiovascular disease. For example, loss of cardiac ferritin H promotes cardiomyopathy SlC7A11‐mediated ferroptosis.^7^ Ferroptosis is a process where phospholipids (PL) containing polyunsaturated fatty acids (PUFA) are excessively oxidized by ferrous iron or ester oxygenases, leading to cell membrane rupture and subsequent cell death.^8^ PUFA are catalysed by acyl‐CoA synthetase long‐chain family member 4 (ACSL4) and lysophosphatidylcholine acyltransferase 3 (LPCAT3) to participate in polyunsaturated fatty acid‐phosphatidylethanolamine (PUFA‐PE) biosynthesis, the primary substrate in lipid peroxidation.^9^, ^10^ Elevated lipid peroxide production damages the normal cell membrane structure and function, ultimately leading to cell death. Glutathione peroxidase 4 (GPX4), an important regulator of lipid peroxidation, inhibits ferroptosis by suppressing lipid peroxidation.^11^, ^12^ GSH depletion leads to GPX4 inactivation, resulting in lipid peroxidation and ROS accumulation, triggering ferroptosis.^13^ Cysteine transport into cells for GPX4 production is mediated by a transport system comprising the SLC7A11 and SLC3A2 subunits.^14^, ^15^ P53 reduces the SLC7A11 expression, affects the cysteine transport function and further inhibits the GPX4 activity, ultimately leading to ferroptosis.^16^, ^17^ Therefore, the effective regulation of the p53/SLC7A11 axis represents an important therapeutic approach for inhibiting ferroptosis.

Sirtuin 1 (Sirt1), a member of the NAD + ‐dependent deacetylase family, plays a crucial role in regulating cellular senescence, inflammation and resistance to oxidative stress.^18^ Sirt1 reduces ZKSCAN3 acetylation, which protects against MPP + ‐induced cell damage.^19^ Polysulfides attenuate diabetic kidney injury by sulfiding Sirt1 to inactivate p65 and the phosphorylation/acetylation of Stat3.^20^ However, Sirt1 also plays a role in promoting inflammation and cell damage by regulating p53. For instance, LARP7 allosterism enhances the ability of Sirt1 to regulate p53 acetylation, inhibiting vascular cell senescence and decelerating the atherosclerosis process.^21^ Resveratrol is a polyphenolic organic compound found in plants, known for its anti‐inflammatory, antioxidant and antitumor properties. The antioxidant mechanism of resveratrol primarily involves Sirt enzyme activation, which is vital for maintaining cardiovascular health.^22^ However, whether resveratrol inhibits ferroptosis in heart failure and improves heart function via the Sirt1/p53 pathway remains unclear. This aspect is explored in this study.

## 2 MATERIALS AND METHODS

### 2.1 Establishment of heart failure model

The aortic arch of mice was coarcted to establish a mouse model of heart failure. Analgesia was performed with preoperative intragastric administration of meloxicam. Mice were anaesthetised by intraperitoneal injection of 0.3% pentobarbital sodium at a dose of 0.25 mL/10 g. Mice were fixed with medical adhesive tape and placed on a constant temperature operating table, which was set at 37°C and disinfected with povidone‐iodine. Ophthalmic scissors were used to incise the skin at the thorax‐neck junction, while the subcutaneous fascia was separated to expose the suprasternal fossa, allowing the observation of the common carotid arteries on both sides of the trachea. The two lobes of the thymus were gently separated using microscopic forceps, allowing a clear view of the aortic arch. The para‐aortic arch tissue was carefully dissected to separate the aortic arch. A 6–0 surgical silk thread was gently passed under the aortic arch, sutured, and tied into a knot. The tissue and skin were sutured layer‐by‐layer, and the wound was disinfected with povidone‐iodine while mice were placed on a warming table for recovery. After awakening, mice were intraperitoneally injected with 5 mg/kg furosemide to prevent acute heart failure. The sham operation group underwent the same procedure, but without ligation of arteries. Penicillin sodium was intramuscularly injected postoperatively to prevent infection. Animal experiments comply with the arrive guidelines and are carried out in accordance with the National Research Council's Guide for the Care and use of Laboratory Animals. This study was approved by the Animal Ethics Committee of First Affiliated Hospital of Bengbu Medical College (approval, byyfy‐kyk‐201,911).

### 2.2 Resveratrol preparation

Resveratrol powder (R8350; Solarbio Biological Company) was first dissolved in DMSO to a concentration of 20 mg/mL. The solution was then filtered through a 0.22 μm pore size filter to remove bacteria and impurities, obtaining a resveratrol stock solution. The stock solution was then mixed with physiological saline to prepare the administered resveratrol solution. Mice were administered resveratrol by oral gavage at a dose of 50 mg/kg/d,^23^ whereas the sham operation group received the same amount of normal saline.

### 2.3 Mice grouping

The heart tissue‐specific *Sirt1* knockout C57BL/6J mice used in this experiment were purchased from Saiye Biological Company (Suzhou, China). Sirt1 [flox−/+] mice crossed with Cre tool mice to obtain heart‐specific Sirt1 knockout mice. Other C57BL/6 mice were purchased from the Animal Experiment Centre of Bengbu Medical College. All animals used for experiments were 2‐month‐old male mice. Mice were divided into a sham operation group (Sham), heart failure group (HF), heart failure + resveratrol group (HF + Res) and KO‐Sirt1 heart failure + resveratrol group (KO‐HF + Res). There were 14 mice per group and each group was housed in separate cages. Mice used in experiments were raised in the same environment for 10 months.

### 2.4 Cardiac ultrasound

Echocardiography was performed using a Vevo1100 small animal ultrasound imaging platform and an MS400 ultrasound probe, with the probe frequency set to 30 MHz. The left ventricular end‐systolic diameter (LVID; s), left ventricular end‐diastolic diameter (LVID; d) and left ventricular ejection fraction (LVEF) were obtained. Three consecutive cardiac cycles were analysed, and the average value was calculated.

### 2.5 Mouse left ventricle weight and myocardium tissue section

At the end of the experiment, mice were euthanized by CO_2_ asphyxiation after weighing. The mouse heart was removed, and the ventricular blood was washed away in phosphate‐buffered saline (PBS) containing heparin. Excess water was blotted with filter paper, while the heart tissue was removed using surgical scissors and the left ventricle was weighed. The body weight (BW), LV weight (LW) and ventricular mass index (BW/LW) were calculated. The apical tissue of the mouse heart was fixed in a 4% paraformaldehyde solution for 48 h. After dehydration, wax soaking and embedding, the heart tissue was cut into 6 μm‐thick slices.

### 2.6 Differentiation of hiPSCs

HiPSCs were purchased from Cellapy Biological Company (Beijing, China). First, the working solution for cell bottoming (CA3003100, Cellapy Biological Company) was used to fully cover the bottom of the culture dish, which was placed in a 37°C incubator overnight. The next day, the bottoming solution was removed without rinsing and hiPSCs were inoculated in the culture dish. When hiPSCs reached approximately 90% confluence, myocardial induction differentiation reagents (CA2004500, Cellapy Biological Company) were added to induce the differentiation of hiPSCs into cardiomyocytes (hiPSC‐CMs). The initial culture medium was discarded, and cells were gently washed once over with D‐PBS, followed by the addition of myocardial differentiation reagent I and culturing at 37°C. After 48 h, the myocardial induction differentiation reagent I was discarded, and cells were gently washed once with D‐PBS, followed by the addition of myocardial induction differentiation reagent II and continued culturing at 37°C. After 72 h, the myocardial induction and differentiation reagent II were discarded, and cells were gently washed once with D‐PBS, followed by the addition of myocardial induction and differentiation reagent III and culturing at 37°C, with changing of the medium every 48 h. Cells were observed until beating cardiomyocytes appeared. At this point, the myocardium purification culture reagent was added to cells that were cultured for another 48 h until purified cardiomyocytes were obtained. See Supplementary Material 1 for specific experimental results.

### 2.7 Isoproterenol and erastin solution preparation

Cell models were established using isoproterenol (Solarbio Biological Company). Isoproterenol was dissolved in DMSO at a concentration of 10 mg/mL to prepare a stock solution. Next, 62 μL of the stock solution was added to 50 mL of medium to prepare 50 μM isoproterenol (ISO) medium for the establishment of the heart failure cell model.^24^ To prepare a stock solution of erastin, 2 mg of erastin was dissolved in 370 μL of DMSO to achieve a concentration of 10 mM. Subsequently, a 10 μM solution was prepared by adding 50 μL of the stock solution to 50 mL of culture medium to be used for cardiomyocyte experiments.^25^

### 2.8 Lentiviral transfection

The Sirt1 interference and P53K382R acetylation mutant viral vectors were used to transfect hiPSC‐CMs. These viral vectors were purchased from ABM Biological Company (Jiangsu, China). Briefly, 6 μg/mL polybrene and viral vector were mixed and cocultured with cells for 48 h, followed by replacement with fresh medium and observation of fluorescence expression. Puromycin was used for the selection of cells after transfection with viral vectors, while western blotting was used to detect the transfection efficiency. The primers used were as follows: 5′‐CCGGGCGGGAATCCAAAGGATAATTCTCGAGAATTATCCTTTGGATTCCCGCTTTTTG (*SIRT1*‐shRNA forward) and 5′‐AATTCAAAAAGCGGGAATCCAAAGGATAATTCTCGAGAATTATCCTTTGGATTCCCGC (*SIRT1*‐shRNA reverse). See Supplementary Material 2 for vector transfection efficiencies.

### 2.9 SOD detection

The SOD detection in cells and tissues was determined using the WST‐8 assay (S0101M, Beyotime Biological Company). For cell samples, cells were collected and washed with 4°C PBS. Next, 200 μL of SOD sample preparation solution was added to every aliquot of 1 × 10^6^ cells, which were lysed by pipetting. For animal tissue samples, each tissue was washed with PBS containing heparin to remove any blood, and then 150 μL of SOD sample preparation solution was added to every 10 mg of tissue. The tissue was homogenized and centrifuged at 12000 × *g* for 5 min, and the supernatant was collected for testing.

### 2.10 Lipid oxidation content detection

To fully lyse cells or tissues, 100 and 200 μL of lysis buffer was added to 1 × 10^6^ cells and 10 mg of tissue, respectively and incubated on ice. The obtained lysate was centrifuged at 10000 × *g* for 10 min and the supernatant was collected for subsequent assays. To measure the levels of MDA, MDA detection working solution (S0131S, Beyontian Biological Company) was added to the supernatant, and heated at 100°C for 15 min, followed by cooling to 25°C and centrifuging at 1000 × *g* for 10 min. Then, 200 μL of the supernatant was transferred to a 96‐well plate and the absorbance at 540 nm was measured using a microplate reader.

### 2.11 Mitochondrial membrane potential detection

Test solutions were prepared using a mitochondrial membrane potential test reagent (C2006, Beyotime Biological Company). Briefly, 50 μL JC‐1 (200 ×) reagent was diluted by adding 8 mL ultrapure water, to which 2 mL JC‐1 staining buffer (5 ×) was added and mixed well, to obtain the JC‐1 staining working solution. The JC‐1 staining working solution was then added to the culture dish, covering the cell surface, and cells were incubated at 37°C for 20 min. During incubation, an appropriate amount of JC‐1 staining buffer (1 ×) was prepared by adding 4 mL of distilled water to 1 mL of JC‐1 staining buffer (5 ×). After incubation at 37°C, the JC‐1 staining working solution was discarded and cells were washed twice with JC‐1 staining buffer (1 ×). Cells were then observed under a fluorescence microscope.

### 2.12 Mitochondrial superoxide detection

To prepare a 5 mM stock solution, 50 μg of MitoSOX Red Mitochondrial Superoxide Indicator (40778ES50, Yeasen Biological Company) was added to 13 μL of DMSO and mixed thoroughly. The 5 mM stock solution was then diluted with PBS to obtain a 5 μM working solution. Cells were covered with the working solution and incubated in the dark at 37°C for 20 min. Subsequently, cells were washed thrice with PBS and observed under a fluorescence microscope.

### 2.13 Intracellular ATP concentration determination

The culture medium was aspirated and 50 μL of lysis buffer was added to each well of a 24‐well plate to lyse the cells. The lysate was centrifuged at 12000 × *g* and 4°C for 5 min, and the supernatant was obtained. The ATP detection reagent was diluted with an ATP detection reagent diluent at a ratio of 1:9 to prepare the ATP detection working solution. Subsequently, 100 μL of ATP detection working solution was added to the detection well and left standing at 25°C for 5 min. The RLU value was determined using a luminometer and the concentration of ATP was calculated as nmol/mg protein (S0027; Beyotime Biological Company).

### 2.14 P53 protein IP

SDS lysis containing protease inhibitors was added to protein samples and lysed on ice for 30 min, followed by centrifugation at 12000 × *g* for 20 min and collection of the supernatant for later use. The antibody (ab26, Abcam) was diluted with 1 × TBST to prepare a 20 μg/mL antibody working solution. Normal IgG of the same antibody species was used to prepare the IgG working solution at the same dilution ratio to remove nonspecific binding or as a negative control. Subsequently, 500 μL antibody working solution was added to Protein A + G magnetic beads, followed by resuspension and incubation for 2 h. Then, 500 μL of 1 × TBST was added, and placed on the magnetic stand, followed by the removal of the supernatant and separation of magnetic beads. For every 500 μL protein sample, 20 μL Protein A + G magnetic beads with antibody adsorption were added, resuspended, and incubated overnight at 4°C. The next day, an SDS‐PAGE loading buffer was used to separate the obtained protein samples, and western blotting was performed.

### 2.15 Data analysis

Data are expressed as the mean ± standard deviation. GraphPad Prism 9.0 and ImageJ software were used for graphical and statistical analyses. A *t*‐test was used to detect differences between the two groups of data. Analysis of variance (anova) was performed to compare multiple groups of data. A *p*‐value < 0.05 was considered statistically significant. Protein–protein interactions (PPI) were searched using the DAVID website (https://david.ncifcrf.gov/) to explain the potential regulation of ferroptosis by Sirt1.

## 3 RESULTS

### 3.1 Erastin inhibited the therapeutic effect of resveratrol

**FIGURE 1:**
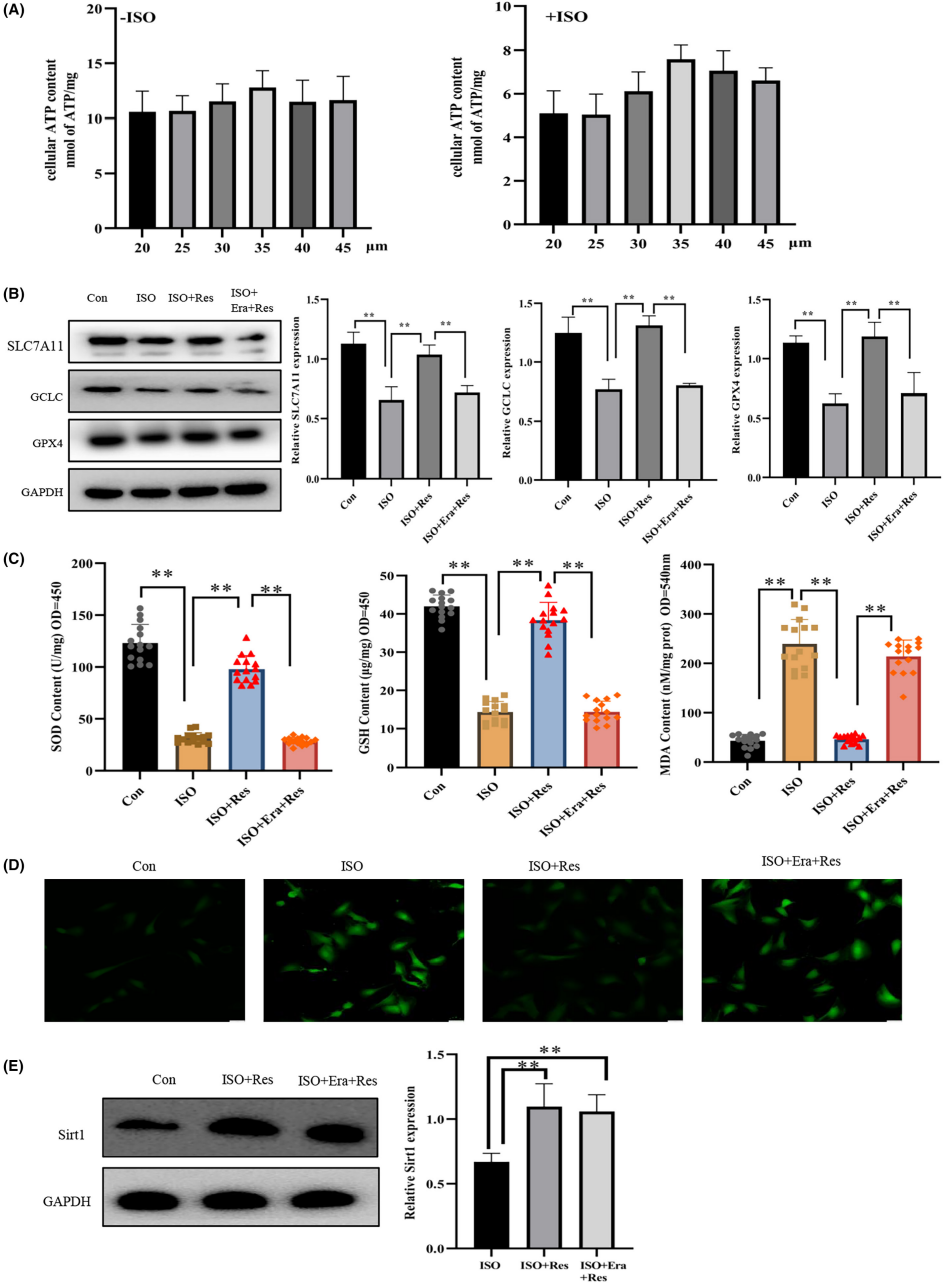
Erastin prohibits resveratrol from inhibiting ferroptosis. (A) An ATP detection kit was used to assess the effects of varying concentrations of resveratrol on cellular viability (*n* = 10/group). (B) Western blot (WB) analysis of the expression of solute carrier family 7 member 11 (SLC7A11), glutathione peroxidase 4 (GPX4) and glutamate‐cysteine ligase catalytic (GCLC) in cardiomyocytes (*n* = 3/group). (C) The WST‐8 method was used to quantify the intracellular levels of SOD, a GSH detection kit was used to assess the intracellular levels of GSH, and a lipid oxidation (MDA) detection kit was used to measure the intracellular levels of lipid oxidation (*n* = 15/group). (D) DCFH‐DA fluorescent probe for detecting the levels of intracellular reactive oxygen species (ROS) (magnification ×20, Scale bar 50 μm). (E) WB detection of the expression of Sirt1 in cells (*n* = 3/group). Con: control group; ISO: isoproterenol‐induced heart failure cell model group; ISO + Res: 35 μM resveratrol treatment group; ISO + Era + Res: 35 μM resveratrol and 10 μM erastin treatment group. Results are means ± SD, **p < 0.001.

The effect of various concentrations of resveratrol on cell viability was evaluated using an ATP detection kit (Figure 1A), and a concentration of 35 μM resveratrol was optimal for treatment. Furthermore, compared to the Con group, ISO treatment resulted in an expression reduction of GPX4, GCLC, and SLC7A11 proteins in cardiomyocytes (Figure 1B). As GPX4, GCLC and SLC7A11 are marker proteins of ferroptosis, a reduction indicated increased ferroptosis.^26^ In contrast, upon the addition of resveratrol, the protein expression was increased (Figure 1B). As intracellular lipid oxidation, ROS accumulation, and decreased levels of SOD and GSH promote ferroptosis, the observed protein expression increase (Figure 1C,D) further suggested that treatment with resveratrol inhibited ferroptosis in cardiomyocytes. However, erastin addition blocked the effects of resveratrol on ferroptosis (Figure 1B–D). Erastin promotes ferroptosis by inhibiting the hydrochloric acid/glutamic acid antiporter (System Xc, in which SLC7A11 is the main constituent protein).^28^ Interestingly, while erastin inhibited the therapeutic effect of resveratrol, it did not alter Sirt1 protein expression (Figure 1E). These findings suggest that resveratrol acts via the Sirt1/System Xc pathway to inhibit ferroptosis progression in cardiomyocytes.

### 3.2 Resveratrol inhibits ferroptosis during early heart failure

**FIGURE 2:**
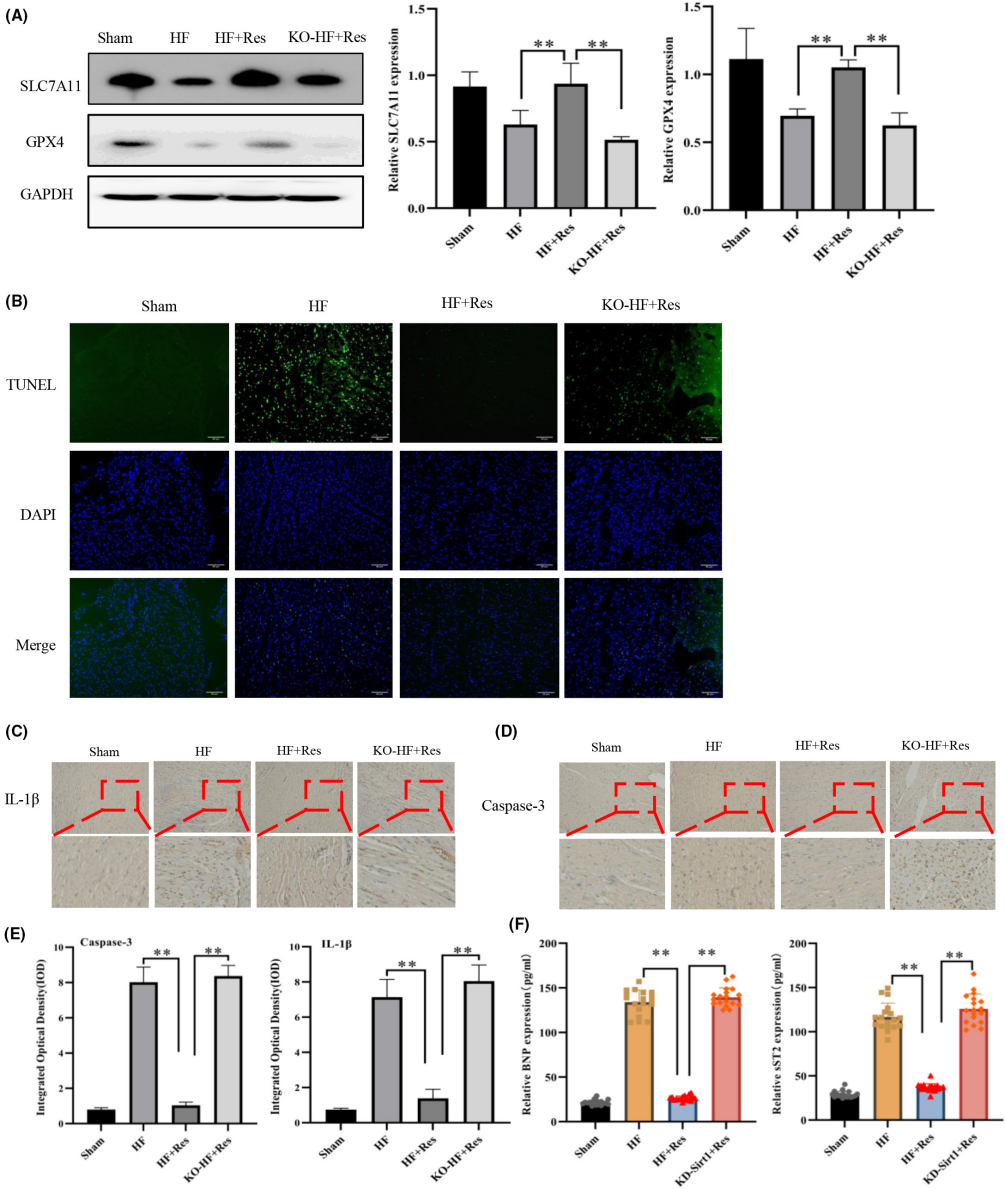
Resveratrol inhibits ferroptosis in the early treatment of heart failure. (A) Western blot (WB) detection of the expression of SLC7A11, GPX4 in myocardial tissues (*n* = 3/group). (B) The level of apoptotic cells in tissues detected by TUNEL (magnification × 20, Scale bar 50 μm). (C, D) Immunohistochemical detection of the expression of IL‐1β and caspase‐3 in myocardial tissues (magnification ×20, Scale bar 50 μm). (E) Quantitation of immunohistochemistry stain of IL‐1β and caspase‐3 in myocardial tissues. (F) Enzyme‐linked immunosorbent assay (ELISA) detection of sST2 and BNP expression levels in the serum of mice (*n* = 18/group). Sham: sham operation group; HF: heart failure group; HF + Res: heart failure + resveratrol group; KO‐HF + Res: KO‐Sirt1 heart failure + resveratrol group. Data represent the mean ± SD for 6 mice in each group, **p < 0.001.

On the 20th day after successful establishment of the HF model, we evaluated the therapeutic potential of resveratrol against early heart failure. The GPX4 and SLC7A11 expression in the heart tissue of the HF group was decreased, as indicated by western blot analysis (Figure 2A). According to the results of TUNEL detection and immunohistochemical analyses (Figure 2B–E), resveratrol reduced the number of apoptotic cardiomyocytes compared with that in the HF group. Of note, BNP and sST2 are frequently used to assess the volume load and function of the heart, serving as specific diagnostic indicators of heart failure.^29^ Resveratrol reduced BNP and sST2 expression in the serum of mice with heart failure, and improved heart function (Figure 2F). However, after knocking out the cardiac *Sirt1* gene, the protective effects of resveratrol, such as ferroptosis inhibition and cardiomyocyte damage deceleration, were lost. These results suggested that in the early stages of heart failure, resveratrol inhibits ferroptosis and enhances the early cardiac function of mice with heart failure in a Sirt1‐dependent manner.

### 3.3 Effect of resveratrol on ferroptosis in long‐term heart failure

**FIGURE 3:**
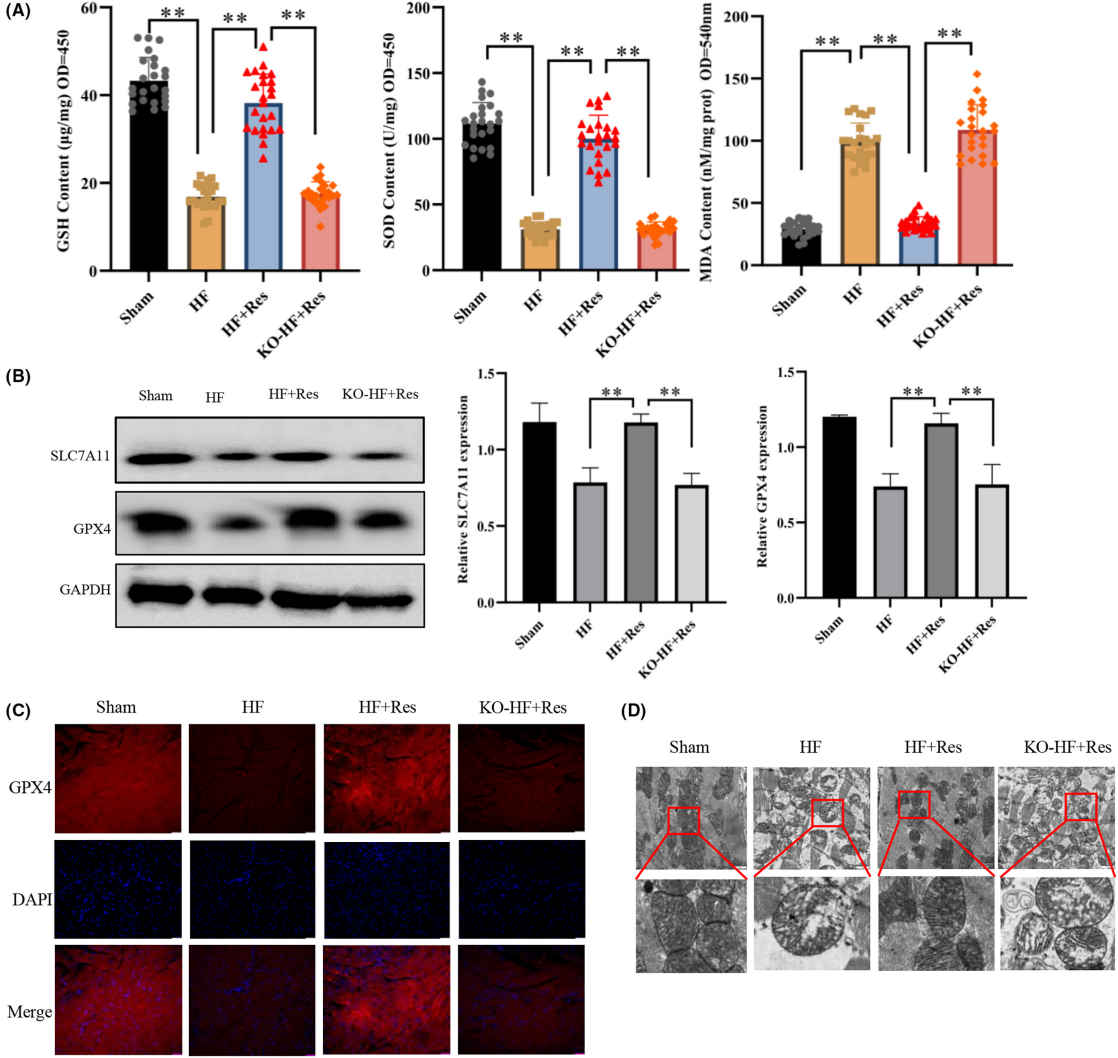
The effect of resveratrol on ferroptosis in long‐term heart failure. (A) Quantification of the intracellular levels of SOD using the WST‐8 method, intracellular GSH levels using a GSH detection kit, intracellular lipid oxidation level determination using a lipid oxidation (MDA) detection kit (*n* = 24/group). (B) Western blot (WB) detection of SLC7A11 and GPX4 expression in myocardial tissues (*n* = 3/group). (C) Indirect immunofluorescence detection of the levels of expression of GPX4 in myocardial tissues (magnification ×20, Scale bar 50 μm). (D)Transmission electron microscopy (TEM) was used to examine mitochondrial morphology in the myocardium (Scale bar 2 μm). Sham: sham operation group; HF: heart failure group; HF + Res: heart failure + resveratrol group; KO‐HF + Res: KO‐Sirt1 heart failure + resveratrol group. Data represent the mean ± SD for 8 mice in each group, **p < 0.001.

To evaluate the long‐term therapeutic effect of resveratrol on heart failure, we performed additional experiments in mice after 10 months. Compared with the HF group, resveratrol lowered the level of fat oxidation. Furthermore, it increased the GSH and SOD expression in the heart tissue of mice (Figure 3A). Resveratrol administration for an extended period led to an increase in GPX4 and SLC7A11 expression in the myocardial tissue, effectively decelerating ferroptosis (Figure 3B,C). As mitochondria are the primary organelles responsible for cellular oxidative phosphorylation and ROS generation, any morphological alterations are indicators of ferroptosis.^27^ The inner mitochondrial membrane had shrunk and the mitochondrial membrane had ruptured in the cardiomyocytes of HF mice. However, resveratrol significantly improved the shape of mitochondria compared with that in the HF group, indicating myocardial ferroptosis suppression (Figure 3D). Conversely, the therapeutic efficacy of resveratrol was ameliorated following knocking‐out of the Sirt1 gene; without significant alterations in mitochondrial morphology and levels of GSH, SOD, or associated proteins in the myocardium compared with the HF group.

### 3.4 Effect of resveratrol on long‐term cardiac function

**FIGURE 4:**
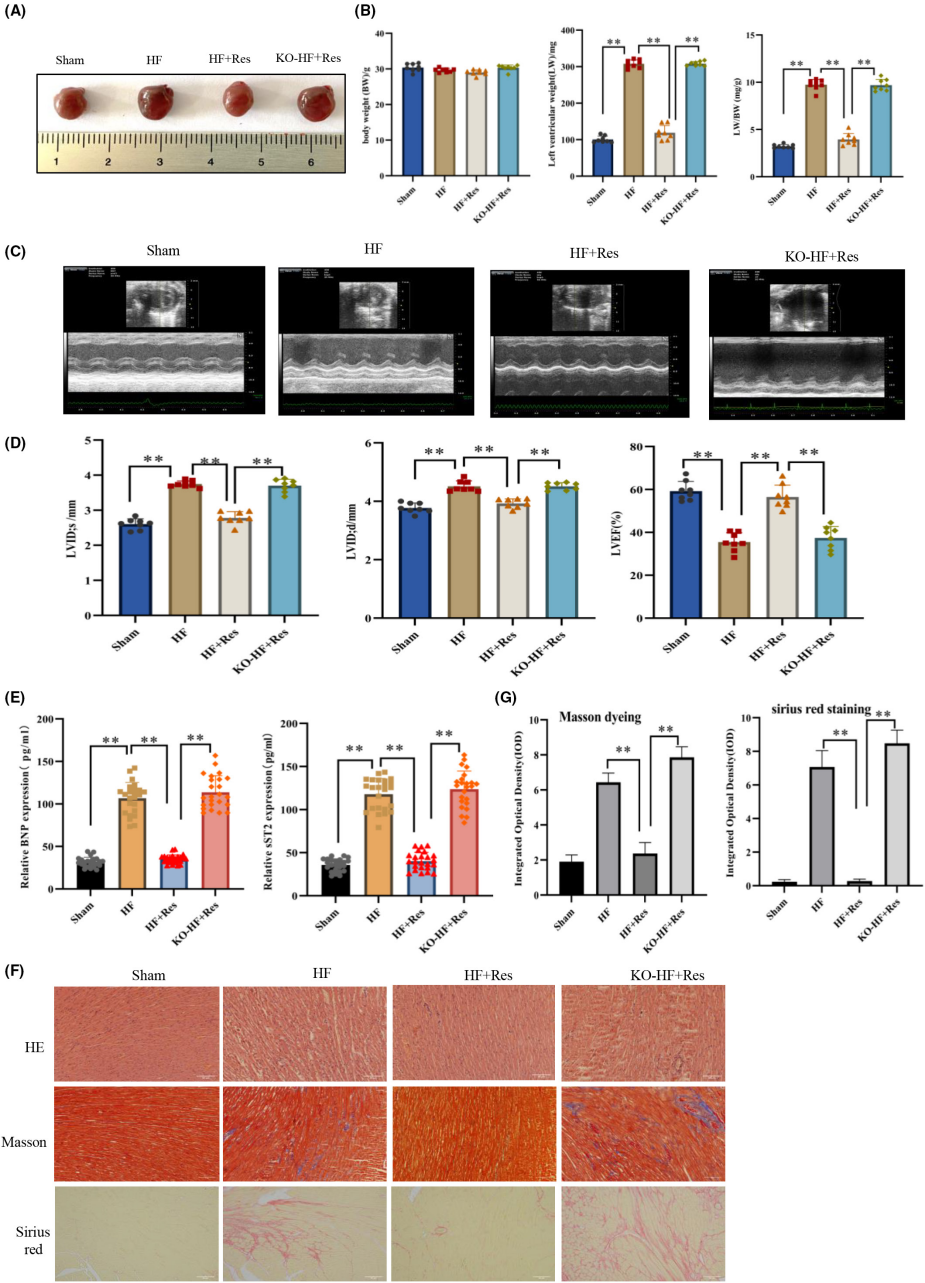
The effects of resveratrol on cardiac function in long‐term heart failure. (A, B) Heart size, body weight (BW), left ventricular weight (LW), and LW/BW measurements (*n* = 8/group). (C) Mouse echocardiographic image results (*n* = 8/group). (D) The left ventricular end‐systolic inner diameter (LVID; s), left ventricular end‐diastolic inner diameter (LVID; d), and left ventricular ejection fraction (LVEF) were measured by echocardiography. (E) Enzyme‐linked immunosorbent assay (ELISA) detection of the sST2 and BNP expression in the serum of mice (*n* = 24/group). (F) Paraffin sections of heart tissue stained with haematoxylin and eosin (HE), Masson's trichrome, and Sirius Red (magnification × 20). (G) The degree of myocardial fibrosis marked by Masson staining and Sirius red staining was quantitatively analysed. Sham: sham operation group; HF: heart failure group; HF + Res: heart failure + resveratrol group; KO‐HF + Res: KO‐Sirt1 heart failure + resveratrol group. Data represent the mean ± SD for eight mice in each group, **p < 0.001.

Following a 10‐month period, resveratrol effectively hindered the heart enlargement observed in mice with heart failure, resulting in reduced LW and LW/BW values (Figure 4A,B). Based on heart failure indicators and echocardiogram assessments, long‐term resveratrol administration safeguarded the left ventricle function and enhanced the cardiac ejection fraction in heart failure in the long term (Figure 4C–E). Furthermore, resveratrol ameliorated myocardial oedema and hypertrophy in the event of long‐term heart failure, while simultaneously reducing the degree of myocardial fibrosis (Figure 4F,G). Interestingly, Sirt1 suppression in the heart tissue impeded the efficacy of resveratrol in inhibiting myocardial fibrosis and enhancing long‐term cardiac function. Hence, the ability of resveratrol to inhibit ferroptosis and exhibit therapeutic benefits in the treatment of advanced heart failure in mice is dependent on Sirt1.

### 3.5 Resveratrol inhibited ferroptosis in hiPSC‐CMs in a Sirt1‐dependent manner

**FIGURE 5:**
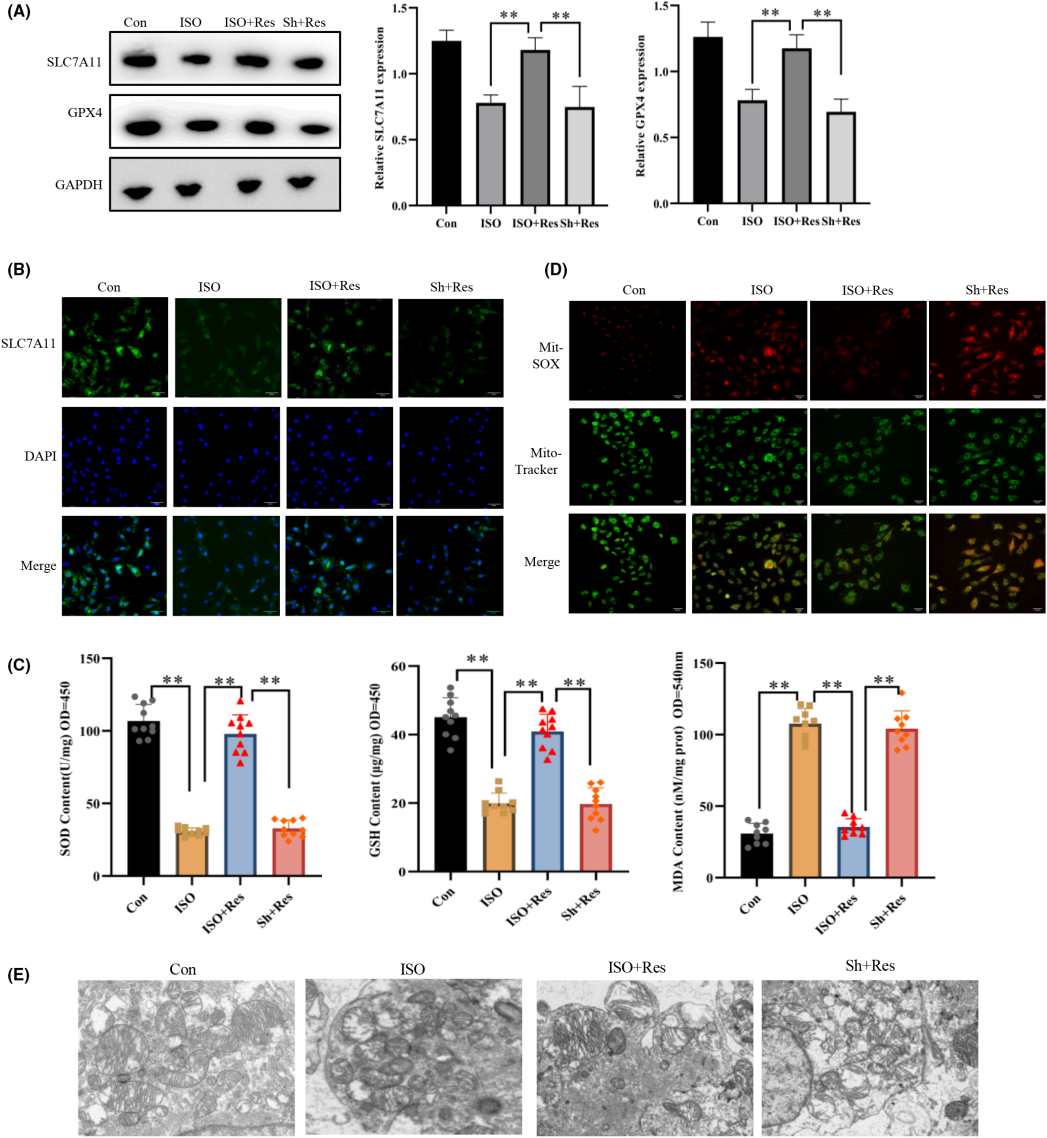
The Sirt1 pathway mediates the protective effect of resveratrol on human‐induced pluripotent stem cell‐derived cardiomyocytes (hiPSC‐CM) damage. (A) WB detection of the expression of SLC7A11 and GPX4 in cardiomyocytes (*n* = 3/group). (B) Indirect immunofluorescence detection of the levels of expression of GPX4 in cardiomyocytes (magnification × 20). (C) Intracellular levels of superoxidase dismutase (SOD) using the WST‐8 method; assessment of the intracellular levels of glutathione (GSH) using a GSH detection kit, and quantification of the intracellular level of lipid oxidation using a lipid oxidation (MDA) detection kit (*n* = 10/group). (D) Superoxide levels in the mitochondria of cardiomyocytes by the MitoSOX Red mitochondrial superoxide indicator (magnification × 20). (E) Mitochondria morphology in the myocardium using transmission electron microscopy (TEM). Con: control group; ISO, isoproterenol‐induced heart failure cell model group; ISO + Res: 35 μM resveratrol treatment group; Sh + Res: transfect Sh‐Sirt1 lentiviral vector + isoproterenol + 35 μM resveratrol. Results are means ± SD, **p < 0.001.

Following ISO induction, the GPX4 and SLC7A1 levels, as well as those of GSH and SOD, were reduced, whereas lipid oxidation was increased in cardiomyocytes (Figure 5A–C). Simultaneously, ISO treatment led to oxide accumulation in the mitochondria, shrinkage of the mitochondrial inner membrane and rupture of the mitochondrial membrane (Figure 5D,E). These changes observed in cardiomyocytes after ISO induction was in line with experimental findings in animal models, supporting the involvement of ferroptosis in the development of heart failure. Conversely, we found that resveratrol mitigated the ISO‐induced ferroptosis in cardiomyocytes, thereby safeguarding cardiomyocytes and inhibiting apoptosis. However, knocking down Sirt1 expression resulted in a loss of ability to inhibit ferroptosis, indicating that Sirt1 is an essential factor in the inhibition of ferroptosis by resveratrol.

### 3.6 Resveratrol increased the expression of SLC7A11 via the Sirt1/p53 pathway

**FIGURE 6:**
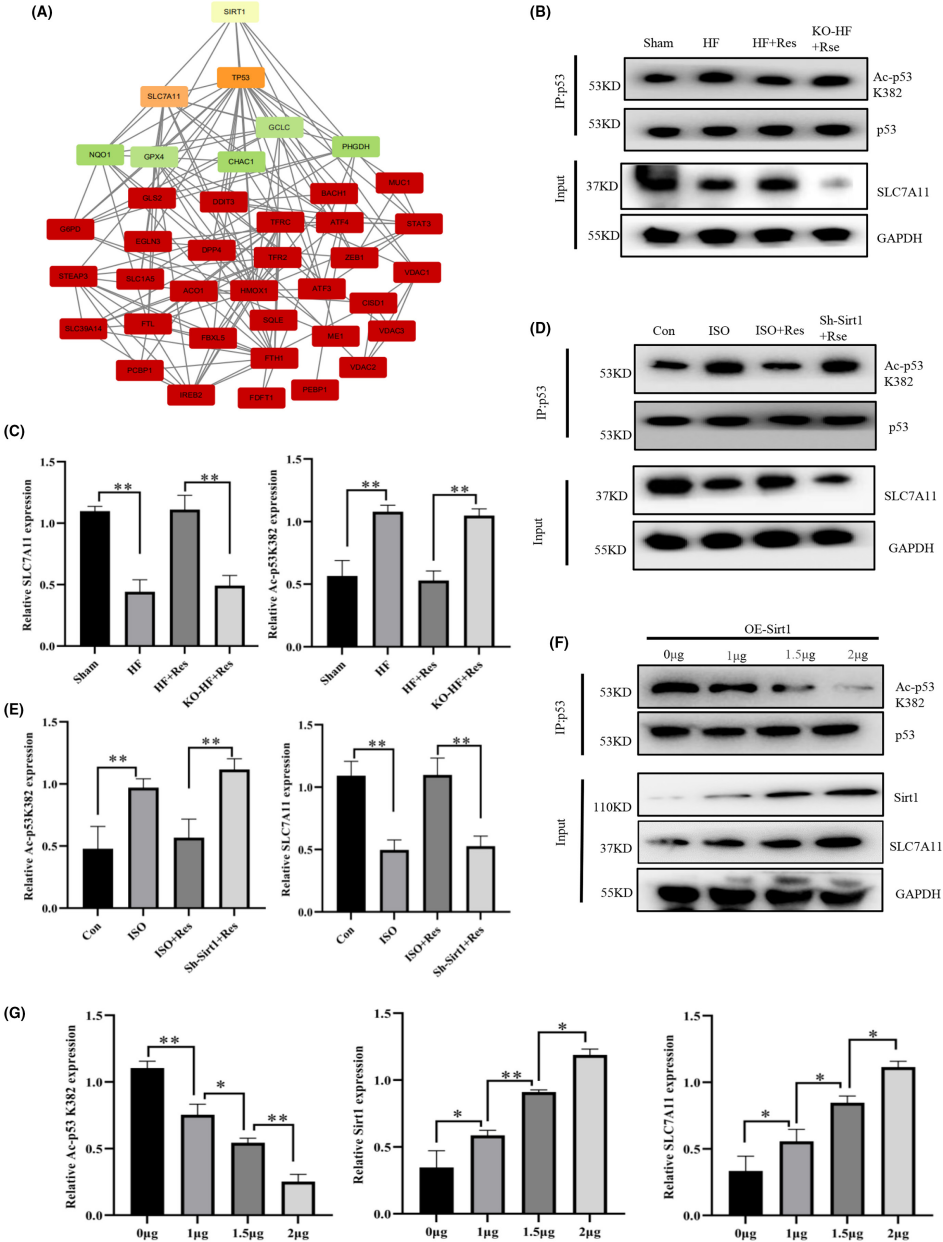
Resveratrol increases the expression of SLC7A11 via the Sirt1/p53 pathway. (A) Protein–protein interaction (PPI) assay indicating the relationship between Sirt1 and ferroptosis‐related proteins. (B, C) Collection of myocardial tissue protein samples for immunoprecipitation (IP) and western blot (WB) analysis (*n* = 3/group). (D, E) Collection of in vitro cardiomyocyte protein samples for IP and WB analysis (*n* = 3/group). (F, G) Human induced pluripotent stem cell‐derived cardiomyocytes (hiPSC‐CMs) were transfected with the indicated plasmids and harvested for IP and immunoblotting with the relevant antibodies (*n* = 3/group). Results are means ± SD, **p < 0.001, *p < 0.05.

Sirt1 functions as a NAD‐dependent deacetylase that can directly deacetylate p53 and modulate its activity. Furthermore, Sirt1 can exert its biological effects by inactivating the Lys382 residue of p53.^30^, ^31^ Using a protein interaction database, we found that Sirt1 plays a role in ferroptosis via the p53/SLC7A11 pathway (Figure 6A). SLC7A11 is a primary component of System Xc, which explains why erastin blocks the inhibitory effects of resveratrol on ferroptosis. We performed IP assays to further analyse the interactions between these proteins and found that resveratrol reduced the levels of p53 K382 acetylation in both animal and cell models. However, after inhibiting Sirt1 expression, resveratrol lost its ability to reduce the levels of p53 K382 acetylation (Figure 6B–E). We then transfected ISO‐treated cells with different concentrations of a Sirt1‐overexpressing plasmid and observed a gradual decrease in the levels of p53 K382 acetylation, whereas an increase in the expression of SLC7A11 with increasing concentration of Sirt1 (Figure 6F,G). Conclusively, resveratrol inhibited ferroptosis by reducing the levels p53 K382 acetylation and increasing SLC7A11 protein expression.

### 3.7 P53 K382 acetylation reduction attenuated cardiomyocyte ferroptosis

**FIGURE 7:**
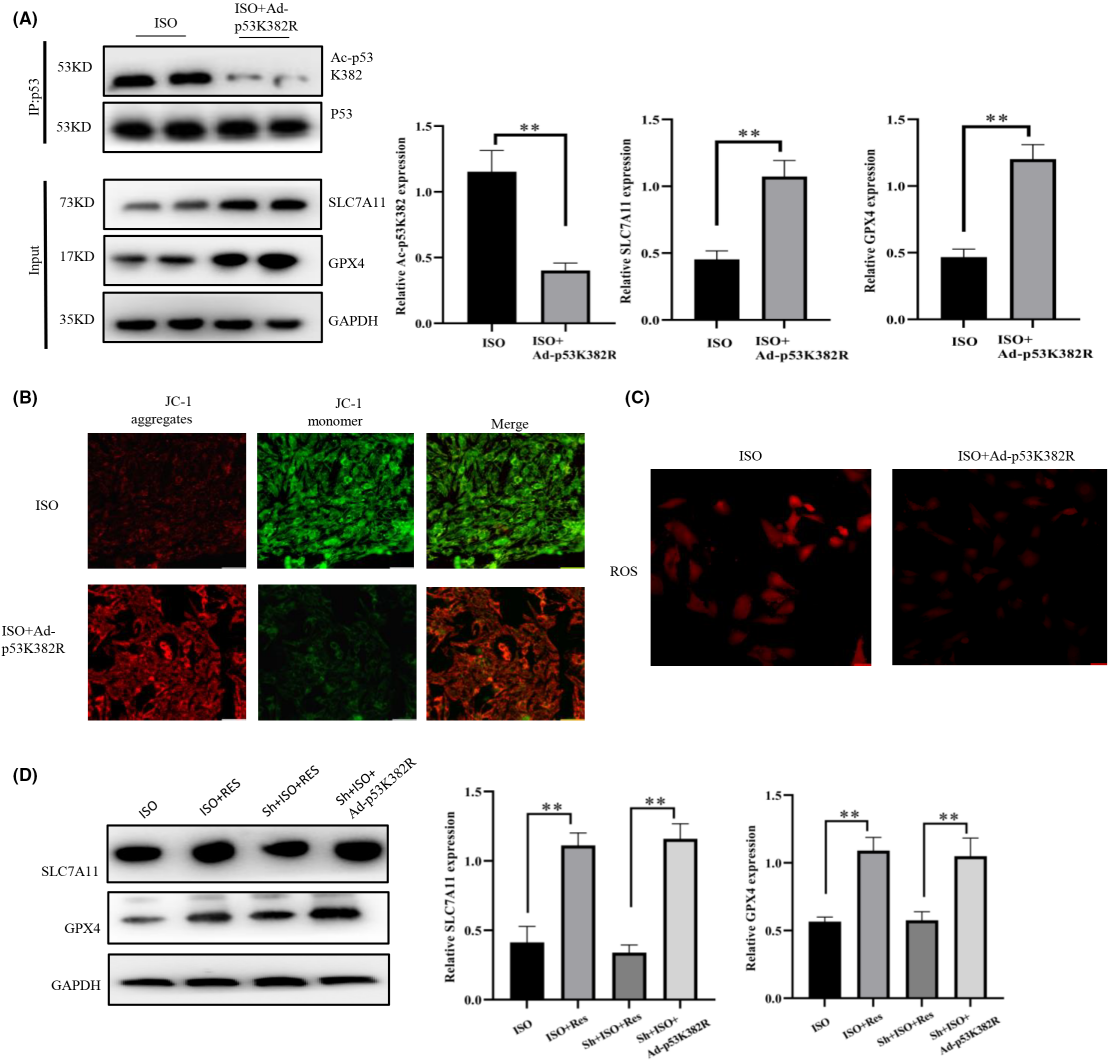
P53 K382 acetylation reduction attenuates cardiomyocyte ferroptosis. (A) Collection of in vitro cardiomyocyte protein samples for immunoprecipitation (IP) and western blot (WB) analyses (*n* = 3/group). (B) Detection of mitochondrial membrane potential in cardiomyocytes (magnification × 20, Scale bar 50 μm). (C) 2′‐7’dichlorofluorescin diacetate (DCFH‐DA) fluorescent probe for the detection of the intracellular levels of reactive oxygen species (ROS) (magnification × 20, Scale bar 50 μm). (D) WB detection of the protein expression of SLC7A11 and GPX4, suggesting that Ad‐p53K382R can rescue ferroptosis caused by a decrease in the levels of Sirt1 (*n* = 3/group). ISO, isoproterenol‐induced heart failure cell model group; ISO + Ad‐p53K382R: isoproterenol+ Ad‐p53K382R lentiviral vector; Sh + ISO + Ad‐p53K382R: isoproterenol + Sh‐Sirt1/Ad‐p53K382R lentiviral vector. Results are means ± SD, **p < 0.001, *p < 0.05.

We transfected cardiomyocytes with the Ad‐p53K382R adenovirus, which carries a mutation of lysine (K) at position 382 to arginine (R), in order to reduce the acetylation level of the p53 K382 site. IP analyses showed that transfection with Ad‐p53K382R downregulated the acetylation level of p53 K382 in ISO‐treated cells (Figure 7A). Compared with the ISO group, transfection with Ad‐p53K382R increased the protein levels of GPX4 and SLC7A11 in cardiomyocytes. The level of mitochondrial membrane potential is an indicator used for assessing the state of mitochondrial and cellular activity. The reduction in p53 K382 acetylation led to an increase in the mitochondrial membrane potential, whereas it decreased intracellular ROS content and enhanced cell viability (Figure 7B,C). Further experiments revealed that the protective effects of resveratrol on the myocardium were ameliorated following the knockdown of Sirt1. However, we noticed that transfection with Ad‐p53K382R rescued ferroptosis caused by the downregulation of Sirt1 (Figure 7D). Therefore, we concluded that resveratrol inhibited ferroptosis via the Sirt1/p53/SLC7A11 pathway.

## 4 DISCUSSION

Ferroptosis plays a crucial role in the progression of various cardiovascular diseases. For instance, Wang et al. demonstrated that dexmedetomidine inhibited ferroptosis via the AMPK/GSK‐3β/Nrf2 pathway, alleviating myocardial damage resulting from ischemia–reperfusion injury.^32^ Therefore, a deeper understanding of the role of ferroptosis in heart failure is essential to develop effective strategies for heart failure prevention and treatment. Here, we established a heart failure model using aortic coarctation surgery. The levels of ferroptosis‐related proteins, lipid oxidation, and ROS were determined, and mitochondrial morphology was evaluated. Our results indicated that ferroptosis was involved in both the early and long‐term development of heart failure. Resveratrol, a natural activator of Sirt1, has important therapeutic value in the management of the cardiovascular disease.^22^ We used in vivo and in vitro experiments to demonstrate the inhibitory effect that resveratrol has on ferroptosis progression in cardiomyocytes of mice with heart failure. Furthermore, resveratrol delayed myocardial fibrosis, and improved cardiac function. However, Sirt1 knock‐out ameliorated the inhibitory effect of resveratrol on cardiomyocyte ferroptosis and its improvement of heart failure in mice. These results suggested that the therapeutic effect of resveratrol on heart failure is mediated by the Sirt1 pathway.

Cysteine (Cys) deficiency, GSH depletion, and GPX4 inactivation have also been shown to promote ferroptosis.^7^, ^33^ Cys is a component of GSH that is supplied by the cysteine‐glutamate antiporter (system Xc). Accordingly, a reduction in the levels of cysteine/glutamate metabolism will reduce the levels and inhibit the activity of GPX.^34^ Erastin is a small molecule that inhibits the cystine/glutamate antiporter, leading to impaired cystine import and inhibition of GSH synthesis.^28^ System Xc is a dimer composed of two subunits, SLC7A11 and SLC3A2, in which SLC7A11 plays a key role in limiting the transport rate.^35^ We found that the inhibitory effect of resveratrol on ferroptosis was blocked by erastin, leading to a decrease in the levels of GSH and GPX4 in the myocardium. Therefore, we concluded that resveratrol affects the activity of the cysteine‐glutamate transporter through Sirt1, hence playing a role in regulating ferroptosis. Following the Sirt1 knock‐out, we noticed that the ability of resveratrol to protect SLC7A11 from degradation was lost. Consequently, the levels of expression of GSH, GCLC, and GPX4 were decreased, whereas those of ROS and lipid oxidation were increased in cardiomyocytes. Therefore, we suggest that resveratrol requires Sirt1 for maintaining the stability of System Xc.

An acetylation defective mutant P53 with 3 mutated lysine residues (K117/161/162R) fails to induce apoptosis and senescence, but still sensitized cells to ferroptosis.^17^, ^36^ Yang et al. found that STAT6 alleviated acute lung injury by regulating the P53/SLC7A11 pathway to inhibit ferroptosis.^37^ Sirt1 has been shown to reduce the acetylation of p53 K382 to prevent cell senescence and atherosclerosis.^21^ Recent studies confirmed that P53 increased the levels of lipid peroxidation and induced ferroptosis by regulating the expression of SLC7A11.^17^ Here, we observed that treatment with resveratrol led to an increase in the expression of Sirt1, which in turn decreased the acetylation level of p53 K382 in cardiomyocytes. However, in vivo and in vitro experiments revealed that Sirt1 expression knockdown abolished the effect of resveratrol on p53 K382 acetylation. In addition, IP experiments demonstrated that an increase in the expression of Sirt1 resulted in a gradual decrease in p53 K382 acetylation and an increase in the expression of SLC7A11. Hence, these findings suggested that resveratrol inhibits the progression of ferroptosis via Sirt1/p53 pathway activation.

To confirm whether p53 K382 acetylation affects the expression of SLC7A11, a mutant p53 K382R viral vector was transfected into cardiomyocytes to decrease the levels of p53 K382 acetylation in cells. Previous studies have demonstrated that inhibition of p53 K382 acetylation prevented the development of ISO‐induced cardiomyocyte ferroptosis. Simultaneously, the reduction in the levels of p53 K382 acetylation alleviated ferroptosis triggered by the decreased expression of Sirt1. Consequently, we demonstrated that Sirt1 regulates the expression of SLC7A11 through p53 K382 acetylation, thus hindering ferroptosis.^38^ This study verified, for the first time, that resveratrol can suppress ferroptosis, delay myocardial fibrosis, and enhance cardiac function via the Sirt1/p53/SLC7A11 pathway. These findings offer a novel perspective on the pharmacological mechanisms of resveratrol and the management of heart failure. Furthermore, we employed hiPSC‐CMs, which are recognized as a proficient model for disease studies and a valuable tool for pharmacological research. HiPSC‐CMs have been employed in several scientific disciplines, including drug discovery, toxicity investigations, and disease modelling.^39^ Therefore, the cell model used in this study can highlight the disease mechanism of heart failure.

## 5 CONCLUSIONS

**FIGURE 8:**
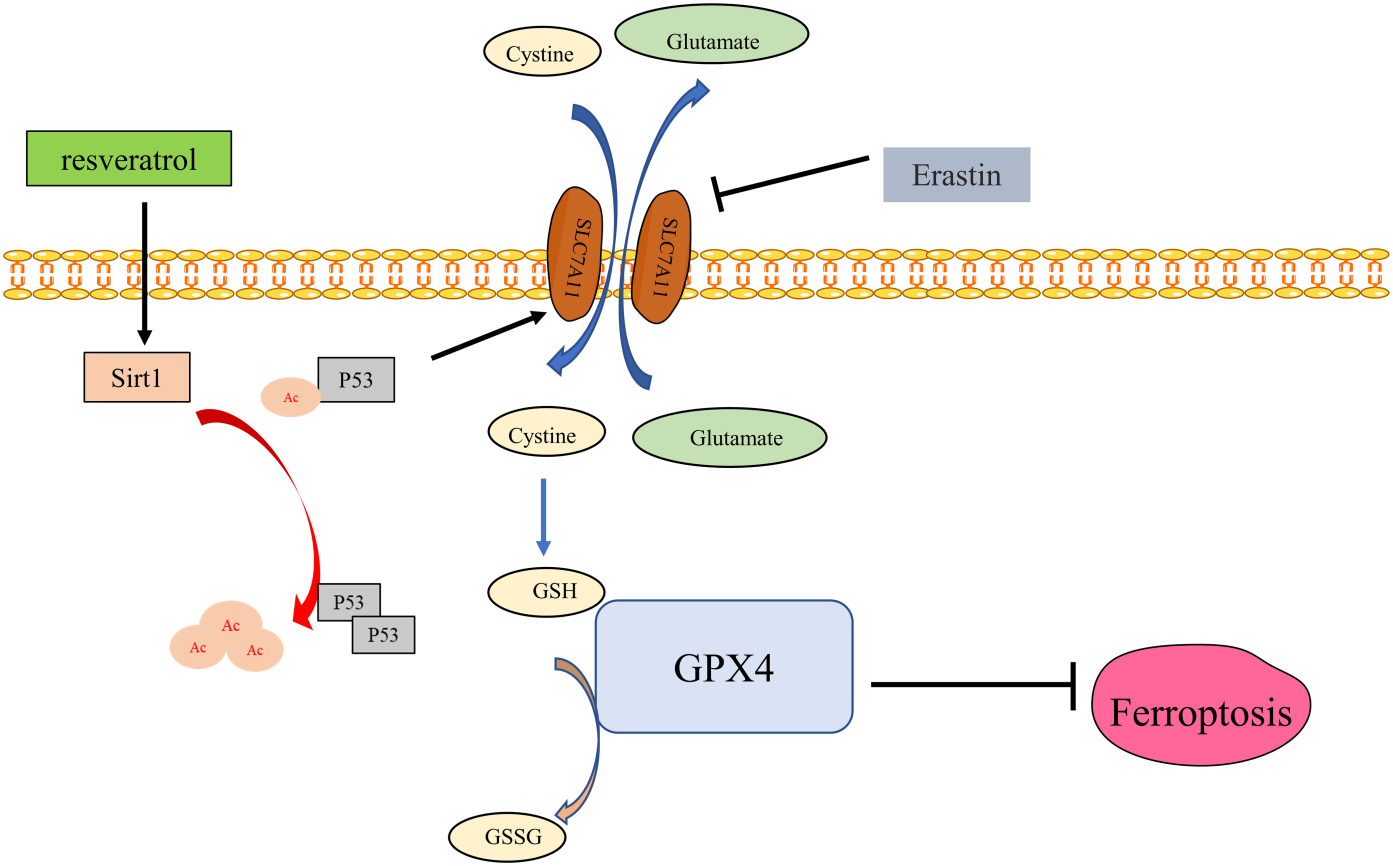
Resveratrol inhibits ferroptosis through the Sirt1/p53 pathway.

This study revealed that by activating Sirt1, resveratrol effectively hindered ferroptosis in heart failure, decelerated the progression of cardiac fibrosis, and enhanced the cardiac function of mice. Particularly, Sirt1 activation led to a decrease in the levels of p53 K382 acetylation, while alleviating the deficiency of SLC7A11 in heart failure. Stabilization of SLC7A11 at a certain level resulted in an increase in the expression of GSH and GPX4 in cells, subsequently suppressing ferroptosis (Figure 8). This discovery opens up a novel approach to the prevention and treatment of heart failure.

## AUTHOR CONTRIBUTIONS

**Wei Zhang:** Conceptualization (equal); investigation (equal); writing – original draft (equal). **Shaohuan Qian:** Data curation (lead); software (supporting); visualization (lead). **Bi Tang:** Data curation (equal); methodology (equal); software (equal); supervision (equal). **Pinfnag Kang:** Data curation (equal); supervision (equal). **Heng Zhang:** Conceptualization (equal); resources (equal); supervision (equal); writing – review and editing (equal). **Chao Shi:** Conceptualization (equal); methodology (equal); project administration (equal); writing – review and editing (equal).

## FUNDING INFORMATION

The present study was supported by research grants from Science and Technology Project of Anhui Province [1804 h08020280].

## CONFLICT OF INTEREST STATEMENT

The author declares that there is no conflict of interest regarding the publication of this paper.

## Supporting information


**Data S1:** Supporting Information

## Data Availability

The author declares that there is no conflict of interest regarding the publication of this paper.The raw data supporting the conclusions of this article will be made available by the authors, without undue reservation.

## References

[1] Boorsma EM , Ter Maaten JM , Voors AA , et al. Renal compression in heart failure: the renal tamponade hypothesis. JACC Heart Fail. 2022;10(3):175‐183. 35241245 10.1016/j.jchf.2021.12.005

[2] von Haehling S , Birner C , Dworatzek E , et al. Travelling with heart failure: risk assessment and practical recommendations. Nat Rev Cardiol. 2022;19(5):302‐313. 34992256 10.1038/s41569-021-00643-z

[3] Groenewegen A , Rutten FH , Mosterd A , Hoes AW . Epidemiology of heart failure. Eur J Heart Fail. 2020;22(8):1342‐1356. 32483830 10.1002/ejhf.1858 PMC7540043

[4] Mascolo A , di Mauro G , Cappetta D , et al. Current and future therapeutic perspective in chronic heart failure. Pharmacol Res. 2022;175:106035. 34915125 10.1016/j.phrs.2021.106035

[5] McDonagh TA , Metra M , Adamo M , et al. 2021 ESC guidelines for the diagnosis and treatment of acute and chronic heart failure. Eur Heart J. 2021;42(36):3599‐3726. 34447992 10.1093/eurheartj/ehab368

[6] Yamamoto T , Sano M . Deranged myocardial fatty acid metabolism in heart failure. Int J Mol Sci. 2022;23(2):996. 35055179 10.3390/ijms23020996 PMC8779056

[7] Fang X , Cai Z , Wang H , et al. Loss of cardiac ferritin H facilitates cardiomyopathy via Slc7a11‐mediated ferroptosis. Circ Res. 2020;127(4):486‐501. 32349646 10.1161/CIRCRESAHA.120.316509

[8] Zhang Y , Xin L , Xiang M , et al. The molecular mechanisms of ferroptosis and its role in cardiovascular disease. Biomed Pharmacother. 2022;145:112423. 34800783 10.1016/j.biopha.2021.112423

[9] Guo Y , Lu C , Hu K , Cai C , Wang W . Ferroptosis in cardiovascular diseases: current status, challenges, and future perspectives. Biomolecules. 2022;12(3):390. 35327582 10.3390/biom12030390 PMC8945958

[10] Stockwell BR , Friedmann Angeli JP , Bayir H , et al. Ferroptosis: a regulated cell death nexus linking metabolism, redox biology, and disease. Cell. 2017;171(2):273‐285. 28985560 10.1016/j.cell.2017.09.021 PMC5685180

[11] Wang Y , Yan S , Liu X , et al. PRMT4 promotes ferroptosis to aggravate doxorubicin‐induced cardiomyopathy via inhibition of the Nrf2/GPX4 pathway. Cell Death Differ. 2022;29(10):1982‐1995. 35383293 10.1038/s41418-022-00990-5 PMC9525272

[12] Wang C , Chen S , Guo H , et al. Forsythoside a mitigates Alzheimer's‐like pathology by inhibiting ferroptosis‐mediated neuroinflammation via Nrf2/GPX4 Axis activation. Int J Biol Sci. 2022;18(5):2075‐2090. 35342364 10.7150/ijbs.69714 PMC8935224

[13] Ursini F , Maiorino M . Lipid peroxidation and ferroptosis: the role of GSH and GPx4. Free Radic Biol Med. 2020;152:175‐185. 32165281 10.1016/j.freeradbiomed.2020.02.027

[14] Lin F , Chen W , Zhou J , et al. Mesenchymal stem cells protect against ferroptosis via exosome‐mediated stabilization of SLC7A11 in acute liver injury. Cell Death Dis. 2022;13(3):271. 35347117 10.1038/s41419-022-04708-w PMC8960810

[15] Ye Y , Chen A , Li L , et al. Repression of the antiporter SLC7A11/glutathione/glutathione peroxidase 4 axis drives ferroptosis of vascular smooth muscle cells to facilitate vascular calcification. Kidney Int. 2022;102(6):1259‐1275. 36063875 10.1016/j.kint.2022.07.034

[16] Stockwell BR , Jiang X . The chemistry and biology of ferroptosis. Cell Chem Biol. 2020;27(4):365‐375. 32294465 10.1016/j.chembiol.2020.03.013 PMC7204503

[17] Jiang L , Kon N , Li T , et al. Ferroptosis as a p53‐mediated activity during tumour suppression. Nature. 2015;520(7545):57‐62. 25799988 10.1038/nature14344 PMC4455927

[18] D'Onofrio N , Servillo L , Balestrieri ML . SIRT1 and SIRT6 signaling pathways in cardiovascular disease protection. Antioxid Redox Signal. 2018;28(8):711‐732. 28661724 10.1089/ars.2017.7178 PMC5824538

[19] Wu X , Ren Y , Wen Y , et al. Deacetylation of ZKSCAN3 by SIRT1 induces autophagy and protects SN4741 cells against MPP+‐induced oxidative stress. Free Radic Biol Med. 2022;181:82‐97. 35124181 10.1016/j.freeradbiomed.2022.02.001

[20] Sun HJ , Xiong SP , Cao X , et al. Polysulfide‐mediated sulfhydration of SIRT1 prevents diabetic nephropathy by suppressing phosphorylation and acetylation of p65 NF‐κB and STAT3. Redox Biol. 2021;38:101813. 33279869 10.1016/j.redox.2020.101813 PMC7718489

[21] Yan P , Li Z , Xiong J , et al. LARP7 ameliorates cellular senescence and aging by allosterically enhancing SIRT1 deacetylase activity. Cell Rep. 2021;37(8):110038. 34818543 10.1016/j.celrep.2021.110038

[22] Tang XL , Wang X , Fang G , et al. Resveratrol ameliorates sevoflurane‐induced cognitive impairment by activating the SIRT1/NF‐κB pathway in neonatal mice. J Nutr Biochem. 2021;90:108579. 33388350 10.1016/j.jnutbio.2020.108579

[23] Chen C , Zou LX , Lin QY , et al. Resveratrol as a new inhibitor of immunoproteasome prevents PTEN degradation and attenuates cardiac hypertrophy after pressure overload. Redox Biol. 2019;20:390‐401. 30412827 10.1016/j.redox.2018.10.021 PMC6226597

[24] Zhao F , Fu L , Yang W , et al. Cardioprotective effects of baicalein on heart failure via modulation of Ca(2+) handling proteins in vivo and in vitro. Life Sci. 2016;145:213‐223. 26706290 10.1016/j.lfs.2015.12.036

[25] Park E , Chung SW . ROS‐mediated autophagy increases intracellular iron levels and ferroptosis by ferritin and transferrin receptor regulation. Cell Death Dis. 2019;10(11):822. 31659150 10.1038/s41419-019-2064-5 PMC6817894

[26] Qiu Y , Cao Y , Cao W , Jia Y , Lu N . The application of ferroptosis in diseases. Pharmacol Res. 2020;159:104919. 32464324 10.1016/j.phrs.2020.104919

[27] Wang F , He J , Xing R , Sha T , Sun B . Molecular mechanisms of ferroptosis and their role in inflammation. Int Rev Immunol. 2023;42(1):71‐81. 34918993 10.1080/08830185.2021.2016739

[28] Riegman M , Sagie L , Galed C , et al. Ferroptosis occurs through an osmotic mechanism and propagates independently of cell rupture. Nat Cell Biol. 2020;22(9):1042‐1048. 32868903 10.1038/s41556-020-0565-1 PMC7644276

[29] Castiglione V , Aimo A , Vergaro G , Saccaro L , Passino C , Emdin M . Biomarkers for the diagnosis and management of heart failure. Heart Fail Rev. 2022;27(2):625‐643. 33852110 10.1007/s10741-021-10105-w PMC8898236

[30] Brooks CL , Gu W . Ubiquitination, phosphorylation and acetylation: the molecular basis for p53 regulation. Curr Opin Cell Biol. 2003;15(2):164‐171. 12648672 10.1016/s0955-0674(03)00003-6

[31] Chien Y , Scuoppo C , Wang X , et al. Control of the senescence‐associated secretory phenotype by NF‐κB promotes senescence and enhances chemosensitivity. Genes Dev. 2011;25(20):2125‐2136. 21979375 10.1101/gad.17276711 PMC3205583

[32] Wang Z , Yao M , Jiang L , et al. Dexmedetomidine attenuates myocardial ischemia/reperfusion‐induced ferroptosis via AMPK/GSK‐3β/Nrf2 axis. Biomed Pharmacother. 2022;154:113572. 35988428 10.1016/j.biopha.2022.113572

[33] Yang Y , Wang Y , Guo L , Gao W , Tang TL , Yan M . Interaction between macrophages and ferroptosis. Cell Death Dis. 2022;13(4):355. 35429990 10.1038/s41419-022-04775-z PMC9013379

[34] Wang L , Liu Y , Du T , et al. ATF3 promotes erastin‐induced ferroptosis by suppressing system xc. Cell Death Differ. 2020;27(2):662‐675. 31273299 10.1038/s41418-019-0380-z PMC7206049

[35] Song X , Zhu S , Chen P , et al. AMPK‐mediated BECN1 phosphorylation promotes ferroptosis by directly blocking system xc‐activity. Curr Biol. 2018;28(15):2388‐2399.e5. 30057310 10.1016/j.cub.2018.05.094 PMC6081251

[36] Li T , Kon N , Jiang L , et al. Tumor suppression in the absence of p53‐mediated cell‐cycle arrest, apoptosis, and senescence. Cell. 2012;149:1269‐1283. 22682249 10.1016/j.cell.2012.04.026 PMC3688046

[37] Yang Y , Ma Y , Li Q , et al. STAT6 inhibits ferroptosis and alleviates acute lung injury via regulating P53/SLC7A11 pathway. Cell Death Dis. 2022;13(6):530. 35668064 10.1038/s41419-022-04971-x PMC9169029

[38] Liu G , Wei C , Yuan S , et al. Wogonoside attenuates liver fibrosis by triggering hepatic stellate cell ferroptosis through SOCS1/P53/SLC7A11 pathway. Phytother Res. 2022;36(11):4230‐4243. 35817562 10.1002/ptr.7558

[39] Okano H , Morimoto S . iPSC‐based disease modeling and drug discovery in cardinal neurodegenerative disorders. Cell Stem Cell. 2022;29(2):189‐208. 35120619 10.1016/j.stem.2022.01.007

