# Supplementary material for: Resveratrol inhibits ferroptosis and decelerates heart failure progression via Sirt1/p53 pathway activation
Source: J Cell Mol Med. 2023 Jul 24;27(20):3075–89. doi: 10.1111/jcmm.17874 (PMC10568670; doi:10.1111/jcmm.17874)
Supplement: Supplementary file 1 — Data S1: Supporting Information [file JCMM-27-3075-s001.docx]

**Supplementary Material**

**Supplementary Material 1**

**Human-derived pluripotent stem cells differentiated into cardiomyocytes**

Microscopic examination revealed a monoclonal cluster of cells with large nuclei, small cytoplasm, and distinct borders (Fig. S1A). Indirect immunofluorescence which high expression of the specific membrane proteins, TRA-1-60 and SSEA, and of the specific transcription factors, NANOG and OCT4 (Fig. S1B). The stemness genes, SOX2, c-Mye, OCT4, and NANOG were also highly expressed in these cells (Fig. S1C). Therefore, the cells used in our study were pluripotent stem cells. We successfully generated beating cardiomyocytes by adding cardiomyocyte differentiation-inducing reagents at the corresponding periods (Fig. S1D). Indirect immunofluorescence analysis revealed the strong expression of cardiac-specific proteins, cTNT, and α-actinin, indicating successful acquisition of hiPSC-CMs (Fig. S1E). Of note, hiPSC-CMs closely resemble human cardiomyocytes in their physiological functions, thereby being able to accurately reflect the efficacy of drugs on human cardiomyocytes.

| 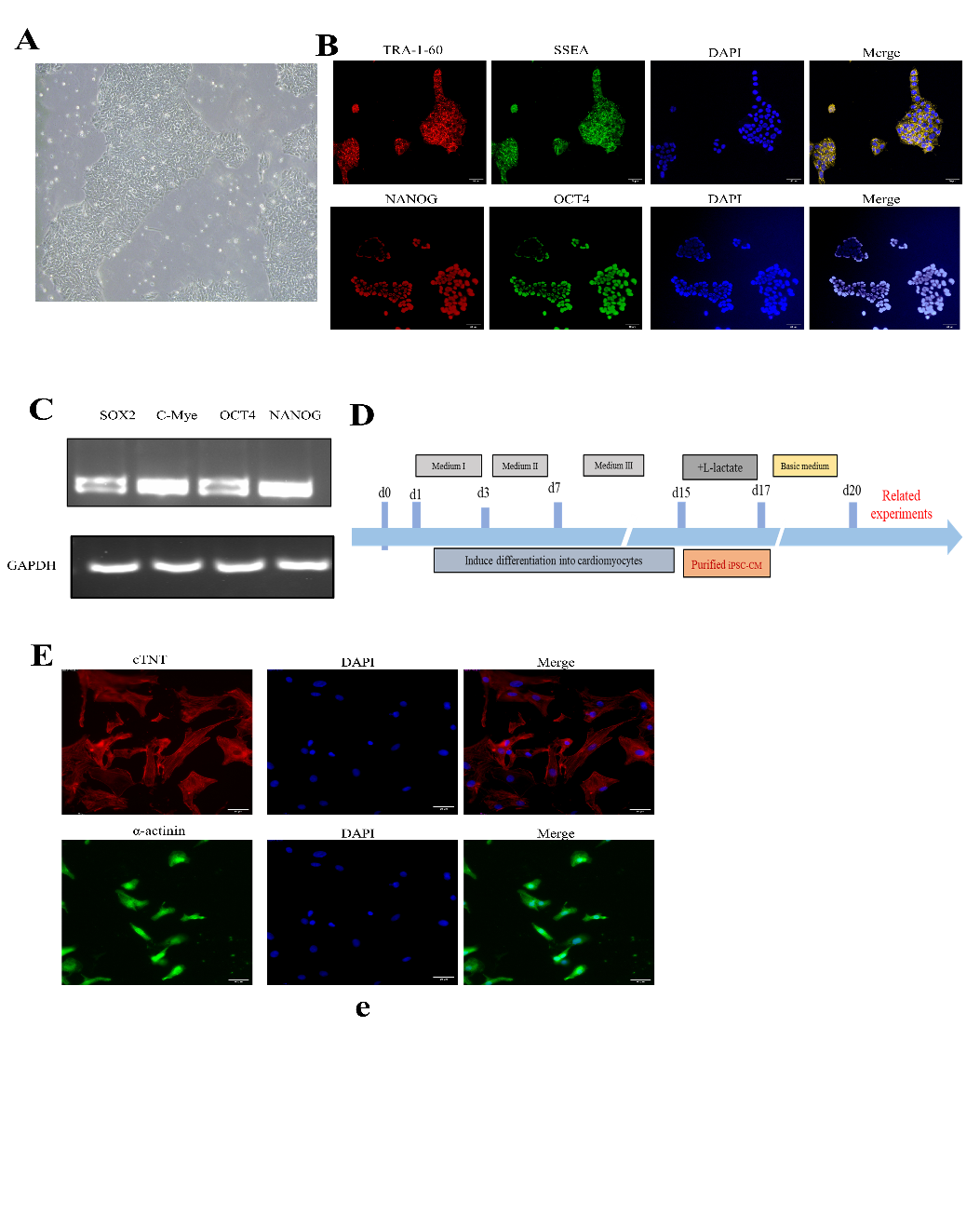 |
| --- |

**S1. Human induced pluripotent stem cell-derived cardiomyocytes (hiPSC) induced differentiation into cardiomyocytes**

A. Monoclonal cell cluster with large nuclei, small cytoplasm, and clean border (magnification ×20). B. Indirect immunofluorescence detection of the stage-specific embryonic antigen (SSEA), T cell receptor alpha locus (TRA-1-60), octamer-binding transcription factor 4 (OCT4), and Nanog expression in cells (magnification ×20, Scale bar 50 μm). C. Polymerase chain reaction (PCR) detection of the stemness genes expression levels, NANOG, SOX2, c-Myc, and OCT4. D. Flowchart of the hiPSC differentiation induction into cardiomyocytes (n=3/group). E. Indirect immunofluorescence detection of the levels of expression of cTNT and α-actinin in cardiomyocytes (magnification ×20, Scale bar 50 μm).

**Supplementary Material 2**

**Validation of viral vector transfection efficiency**

| 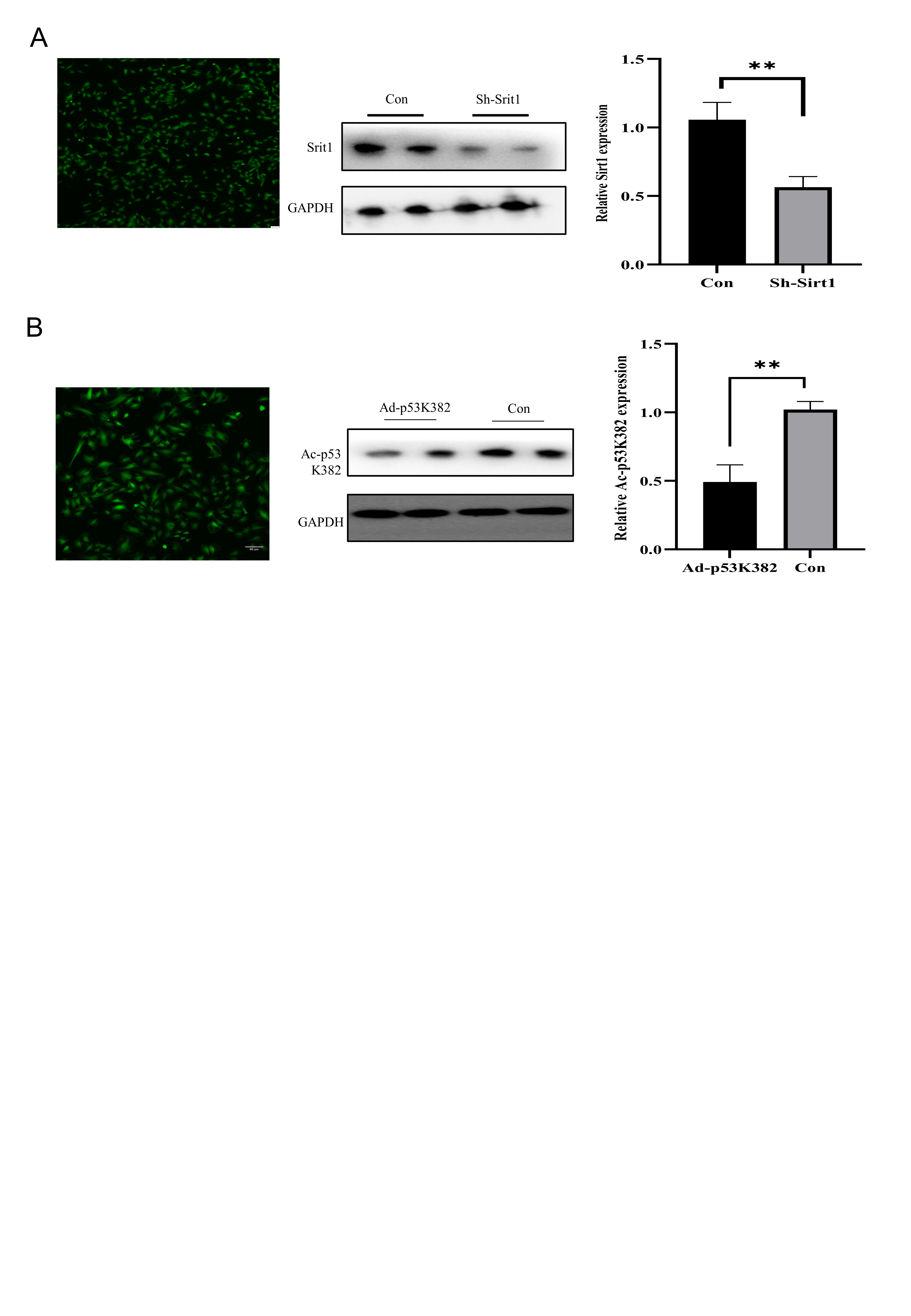 |
| --- |

**S2.Validation of viral vector transfection efficiency**

A. Fluorescence and western blot (WB) detection of Sh-Sirt1 adenovirus transfection efficiency.

B. Verification of the Ad-p53K382R adenovirus transfection efficiency.
